# A Rare Case of Renal Thrombotic Microangiopathy and Focal Segmental Glomerulosclerosis Secondary to Plasma Cell Leukemia

**DOI:** 10.1155/2023/7803704

**Published:** 2023-02-18

**Authors:** Justin Komisarof, Jessica Forman, Bruce Goldman, Chauncey Syposs, Frank Passero, Ellie Garbade

**Affiliations:** ^1^Department of Medicine, University of Rochester Medical Center, 601 Elmwood Avenue, Box MED, Rochester, NY 14642, USA; ^2^Department of Pathology and Laboratory Medicine, University of Rochester Medical Center, 601 Elmwood Avenue, Box 626, Rochester, NY 14642, USA; ^3^Department of Hematology and Oncology, Wilmot Cancer Institute, 601 Elmwood Avenue Box 704, Rochester, NY 14642, USA

## Abstract

Plasma cell dyscrasias are a subset of hematological malignancies involving the production of monoclonal immunoglobulins. This spectrum of disorders includes asymptomatic conditions such as monoclonal gammopathy of unknown significance as well as extremely aggressive malignancies such as plasma cell leukemia. Monoclonal gammopathies are occasionally associated with renal failure, which can occur via many pathophysiological processes. The most common of these is light chain cast nephropathy, but many rare renal complications exist, including thrombotic microangiopathy (TMA) and focal segmental glomerulosclerosis (FSGS). Here, we report a patient with new renal failure with features of TMA and FSGS on biopsy and found to be secondary to plasma cell leukemia.

## 1. Introduction

Multiple myeloma is a plasma cell dyscrasia that is typically characterized by one or more of the “CRAB” criteria (hypercalcemia, renal failure, anemia, and lytic bone lesions) in addition to a bone marrow biopsy with >10% monoclonal plasma cells [1, 2]. A rare and highly aggressive subtype of myeloma is plasma cell leukemia, which is diagnosed when a significant percentage of plasma cells is present in peripheral blood [3, 4]. When plasma cell leukemia arises de novo, it is called primary plasma cell leukemia, whereas when it arises from relapsed/refractory myeloma, it is referred to as secondary plasma cell leukemia [3]. Primary plasma cell leukemia is usually more responsive to therapies and is associated with improved survival compared to secondary plasma cell leukemia [4].

Renal failure is a common manifestation of multiple myeloma, with an incidence of 20–50% on initial diagnosis [5]. The most frequent mechanism of renal injury in multiple myeloma is light chain cast nephropathy, in which light chains form obstructing casts in the distal convoluted tubule [5, 6]. AL amyloidosis is another common cause of renal injury, in which monoclonal light chains form amyloid deposits in the glomerulus, typically resulting in nephrotic range proteinuria [7]. In monoclonal Ig deposition disease, immunoglobulins are deposited along the glomerular basement membrane, also resulting in proteinuria [7, 8]. However, myeloma features rarer mechanisms of renal injury, including thrombotic microangiopathy (TMA) and focal segmental glomerulosclerosis [8, 9].

TMA has been described in multiple myeloma and can be caused by therapies with the proteosome inhibitors carfilzomib or bortezomib [10]. However, TMA has also been documented in patients with monoclonal gammopathies who have not received these therapies [11]. These cases typically feature renal failure, but peripheral findings including microangiopathic hemolytic anemia (MAHA) are not always present [11]. A retrospective study of 146 patients with TMA identified undiagnosed monoclonal gammopathy in 14% of the subjects, far greater than expected [12]. This suggests that the monoclonal gammopathy may contribute to the development of renal TMA. The pathophysiology of FSGS in multiple myeloma has not been studied to a significant degree although the phenomenon has been described in multiple case studies [9, 13].

## 2. Case Report

A 72-year-old female with no significant past medical history presented to the Strong Memorial Hospital Emergency Department (ED) with one month of progressive dyspnea on exertion. She did not take any prescription medications or over-the-counter supplements. A week prior to her ED presentation, she was noted to be newly hypertensive at her primary care physician (PCP)'s office. Shortly, she developed bipedal edema, at which point she was sent to the ED for expedited workup.

**Figure 1:**
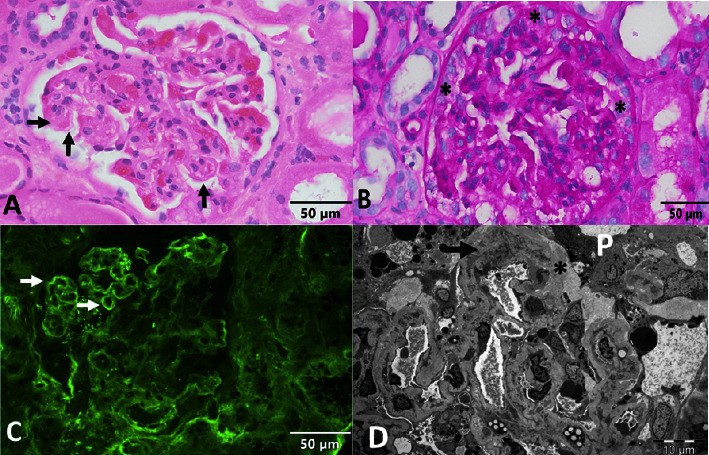
Renal biopsy revealing TMA and FSGS. (a) Light microscopy of the glomerulus with acute thrombotic occlusion in the capillary wall (horizontal arrow) and capillaries with double contours (vertical arrows; hematoxylin and eosin (H&E); original magnification 400×). (b) Light microscopy of the glomerulus with features of collapsing glomerulopathy including capillary collapse and epithelial enlargement and proliferation (asterisks; periodic acid/Schiff base (PAS); original magnification 400×). (c) Immunofluorescence microscopy for fibrin showing the glomerular segment with fibrin deposition in capillary walls (arrows; original magnification 400×). (d) Electron micrograph showing detached podocytes (P) with subepithelial deposition of loose matrix (asterisks) and capillary with mesangial interposition (arrow; original magnification 1500×).

In the ED, she was hypertensive and mildly tachycardic. Initial labs were notable for new renal injury with creatinine of 2.48 mg/dL from a baseline of 0.96 mg/dL one year prior. Her estimated glomerular filtration rate (eGFR) was 20. She was hypoalbuminemic to 2.4 g/dL, but her total protein was elevated at 8.4 g/dL. Urinalysis revealed 2+ blood and 4+ protein. Urine protein-to-creatinine ratio was 16.64. She had a normocytic anemia with hemoglobin of 8.3 g/dL and thrombocytopenia to 110,000/*μ*L. A renal ultrasound noted bilateral increased cortical echogenicity consistent with medical renal diseases without evidence of hydronephrosis. The right kidney was 10 cm in diameter, and the left kidney was 9.9 cm in diameter. She underwent renal biopsy, which was consistent with TMA as well as collapsing FSGS (Figure 1). The biopsy was negative for cast nephropathy, monoclonal immune deposition, and amyloid. Hemolysis labs showed elevated lactate dehydrogenase of 334 U/L and serum haptoglobin of <20 mg/dL, but total bilirubin was normal at 0.2 mg/dL and no schistocytes were seen on peripheral smear.

**Figure 2:**
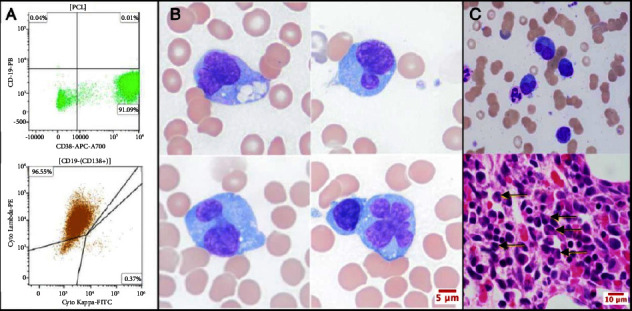
Bone marrow biopsy and peripheral blood flow cytometry revealing plasma cell leukemia. (a) Flow cytometry of peripheral blood demonstrates a discrete population of CD38 positive plasma cells with aberrant loss of CD19; this population of cells is also positive for CD138 and is lambda restricted. (b) Light microscopy of peripheral blood demonstrates leukemic plasma cells with a bizarre bilobed and multilobed nuclear phenotype (original magnification 100×). (c) Light microscopy of bone marrow biopsy demonstrates atypical plasma cells with similar bilobed nuclear morphology (arrows; original magnification 60×).

She was found to have an elevated IgG level at 4576 mg/dL. Lambda free light chains (FLCs) were elevated at 426 mg/dL while kappa FLCs were 1.02 mg/dL. Kappa/lambda FLC ratio was 0.002. An M-spike was present on serum protein electrophoresis with paraprotein of 3.3 g/dL. A skeletal survey showed no lytic lesions. A bone marrow biopsy was performed which in conjunction with peripheral blood flow cytometry was diagnostic for plasma cell leukemia with 10% circulating plasma cells (Figure 2).

She was started on antineoplastic therapy, with cyclophosphamide (300 mg/m^2^ days 1, 8, and 15), bortezomib (1.3 mg/m^2^ days 1, 4, 8, and 11), dexamethasone (40 mg days 1, 4, 8, and 11) (CyBorD), and daratumumab (16 mg/kg weekly) for urgent cytoreduction. She had reduction in circulating plasma cells, serum lambda FLC, and paraprotein. She was discharged from the hospital. After two cycles of chemotherapy, she achieved at least a very good partial response with normalization of serum lambda FLC and unquantifiable paraprotein. Her creatinine remained stable at 2.61 mg/dL with eGFR of 19 and protein-to-creatinine ratio improved to 8.28.

## 3. Discussion

In this report, we describe a case of new renal failure secondary to renal TMA and FSGS in the setting of plasma cell leukemia. This was a challenging diagnosis to make. The initial urinalysis showed hematuria and nephrotic range proteinuria which prompted an expedited renal biopsy to rule out a rapidly progressive glomerulonephritis. The patient displayed the clinical triad of anemia, thrombocytopenia, and renal failure characteristic of MAHA and TMA [14]. However, schistocytes were absent on her peripheral smear, consistent with a renal-limited TMA. Despite the patient's hypoalbuminemia, her total protein was elevated, a “protein gap” that is often indicative of hypergammaglobulinemia [15]. This finding led to a bone marrow biopsy early in the hospital course out of concern for a plasma cell dyscrasia. The combination of renal and bone marrow biopsies clinched the diagnosis.

The etiology of the patient's renal failure was multifactorial. Her initial labs were consistent with nephrotic syndrome. Renal biopsy revealed TMA as well as collapsing-variant FSGS. An association between collapsing FSGS and plasma cell dyscrasias has been reported although the pathophysiology remains unknown [9]. TMA is well-characterized in multiple myeloma, and several mechanisms have been proposed, including Von Willebrand factor dysfunction and uncontrolled complement activation [10, 11, 16]. This case illustrates that monoclonal gammopathies can feature many different renal pathologies and that renal biopsy can be useful in differentiating between these. Notably, if a renal biopsy had not been performed due to the assumption that the patient's renal dysfunction was caused by light chain cast nephropathy, our diagnosis could not have been made.

Due to the patient's aggressive malignancy, the decision was made to initiate chemotherapy while inpatient. While initially the plan was to start CyBorD alone as induction chemo, once the diagnosis of plasma cell leukemia was made, daratumumab was added. Carfilzomib was also considered but not pursued after an echocardiogram revealed wall motion abnormalities and mildly reduced ejection fraction. Despite the known link between carfilzomib and TMA [10], there is currently no evidence on whether this agent should be contraindicated in cases of preexisting TMA. As the link between plasma cell dyscrasias and TMA becomes better understood, new treatment opportunities may become available.

## Data Availability

The imaging, laboratory, and pathologic data used to support the findings of this case study are included within the article.
